# Immunotherapy for glioblastoma: current state, challenges, and future perspectives

**DOI:** 10.1038/s41423-024-01226-x

**Published:** 2024-10-15

**Authors:** Yang Liu, Fei Zhou, Heba Ali, Justin D. Lathia, Peiwen Chen

**Affiliations:** 1 Department of Cancer Biology, Lerner Research Institute, Cleveland Clinic, Cleveland, OH 44195 USA; 2 Department of Neurological Surgery, Feinberg School of Medicine, Northwestern University, Chicago, IL 60611 USA; 3 Department of Cardiovascular and Metabolic Sciences, Lerner Research Institute, Cleveland Clinic, Cleveland, OH 44195 USA; 4 Cleveland Clinic Lerner College of Medicine of Case Western Reserve University, Cleveland, OH 44195 USA; 5 Rose Ella Burkhardt Brain Tumor & Neuro-Oncology Center, Cleveland Clinic, Cleveland, OH 44195 USA; 6 Case Comprehensive Cancer Center, Cleveland, OH 44195 USA

**Keywords:** Glioblastoma, Immune checkpoint inhibitors (ICIs), Adoptive T-cell therapies, Tumor vaccines, Oncolytic viral therapies, Immunotherapy combination, CNS cancer, Immunotherapy

## Abstract

Glioblastoma (GBM) is an aggressive and lethal type of brain tumor in human adults. The standard of care offers minimal clinical benefit, and most GBM patients experience tumor recurrence after treatment. In recent years, significant advancements have been made in the development of novel immunotherapies or other therapeutic strategies that can overcome immunotherapy resistance in many advanced cancers. However, the benefit of immune-based treatments in GBM is limited because of the unique brain immune profiles, GBM cell heterogeneity, and immunosuppressive tumor microenvironment. In this review, we present a detailed overview of current immunotherapeutic strategies and discuss the challenges and potential molecular mechanisms underlying immunotherapy resistance in GBM. Furthermore, we provide an in-depth discussion regarding the strategies that can overcome immunotherapy resistance in GBM, which will likely require combination therapies.

## Introduction

Glioblastoma (GBM), representing approximately half of all primary central nervous system (CNS) malignancies, is the most common primary malignant brain tumor in adults, with an annual incidence of approximately 3 cases per 100,000 people [1]. The standard of care (SOC) therapy for GBM includes maximum safe surgical tumor resection followed by radiotherapy (RT) with concurrent and adjuvant temozolomide (TMZ) chemotherapy [2]. Despite these aggressive treatments, the median overall survival (mOS) of GBM patients remains dismally low, typically ranging from 12–18 months postdiagnosis [3]. The treatment outcomes have remained largely unchanged in recent decades, and most GBM patients experience tumor recurrence. The unique location of GBM tumors in a crucial organ characterized by an immunosuppressive tumor microenvironment (TME) and physical shielding by the blood‒brain barrier (BBB) represents major challenges for GBM treatment and drug delivery [4]. Furthermore, although combination treatment with RT and TMZ can improve the survival of GBM patients [5], this strategy may also trigger TME remodeling to foster a resistant and invasive tumor phenotype [6, 7]. Therefore, novel and effective therapeutic approaches are urgently needed to address these problems and improve GBM patient outcomes.

Cancer immunosurveillance refers to the ability of the immune system to identify and eliminate cancer cells [8]. However, some tumors can evade immunosurveillance through a variety of mechanisms, such as downregulating the expression of tumor antigens and major histocompatibility complex (MHC) molecules, expressing immune inhibitory proteins, and accumulating specific metabolites [9]. In recent years, the field of cancer treatment has undergone significant advancements, with breakthroughs in the development of innovative immunotherapies that strategically modulate the immune system to target tumors [10]. This approach aims to promote cancer eradication by overcoming tumor immunoresistance and offers sustained clinical benefits. Notably, immunotherapy has been shown to eliminate tumors in some types of solid tumors (e.g., melanoma and non-small cell lung cancer) and is recommended as part of the SOC for these tumors [11, 12]. However, the impact of immunotherapy varies across cancer types because of the differences in intrinsic tumor characteristics and the immunosuppressive TME. With respect to GBM, despite limited clinical success, immunotherapy remains a crucial area of ongoing investigation in the field. The promising results of several early clinical trials in GBM and the success in other cancer types offer hope for the development of novel and effective immunotherapies for GBM patients. In this review, we present a detailed overview of the current immunotherapeutic strategies for GBM treatment and discuss the challenges and potential resistance mechanisms of immunotherapy in GBM. Furthermore, we provide an in-depth summary of the strategies that can overcome immunoresistance and improve the antitumor efficacy of immunotherapy in GBM, with a special emphasis on combination treatment options.

## Current immunotherapy state for GBM

**Fig. 1:**
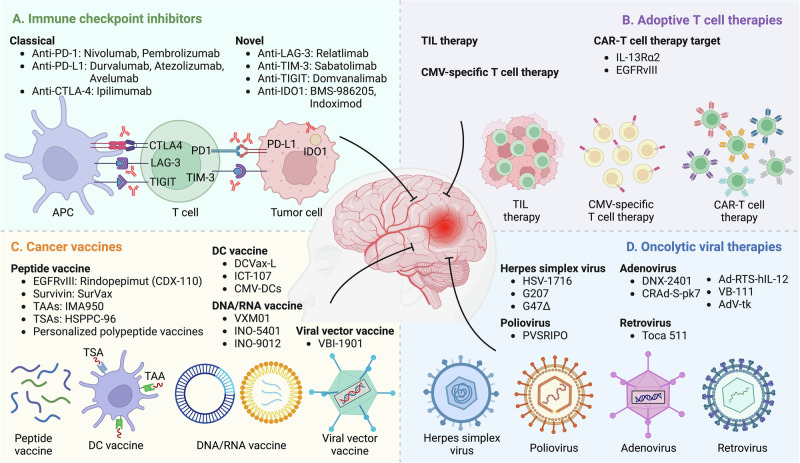
Immunotherapeutic strategies for GBM treatment. Four main immunotherapeutic strategies (immune checkpoint inhibitors, adoptive T-cell therapies, cancer vaccines, and oncolytic viral therapies) have been developed for GBM. **A** Immune checkpoint inhibitors are monoclonal antibodies that target classical (e.g., PD-1, PD-L1, and CTLA-4) and novel (e.g., LAG-3, TIM-3, TIGIT, and IDO1) immune checkpoints. **B** Adoptive T-cell therapy involves the infusion of activated (TILs) or engineered (CMV-specific T cells and CAR-T cells) autologous T cells to increase their antitumor activity. **C** Cancer vaccines use tumor antigens to activate the adaptive immune system of GBM patients, which can be delivered in the form of peptides, DCs, DNA/RNA, and viral vectors. **D** Oncolytic viral therapies (e.g., HSVs, poliovirus, adenovirus, and retrovirus) use replication-competent viruses to selectively infect and destroy cancer cells. Abbreviations: CAR chimeric antigen receptor, CMV cytomegalovirus, CTLA-4 cytotoxic T lymphocyte associated protein 4, DC dendritic cell, EGFRvIII epidermal growth factor receptor variant III, GBM glioblastoma, HSPPC-96 heat shock protein peptide complex-96, IDO1 indoleamine 2,3-dioxygenase 1, IL-13Rα2 interleukin-13 receptor subunit alpha 2, LAG-3 lymphocyte activation gene-3, PD-1 programmed cell death protein 1, PD-L1 programmed cell death ligand 1, TAAs tumor associated antigens, TIGIT T-cell immunoreceptor with immunoglobulin and ITIM domain, TILs tumor infiltrating lymphocytes, TIM-3 T-cell immunoglobulin and mucin domain 3, TSAs tumor specific antigens

The immune system plays an important role in GBM progression, and a range of different immunotherapeutic approaches have been developed to treat GBM patients [13]. In this section, we discuss the current immunotherapeutic strategies designed for GBM, which include immune checkpoint inhibitors (ICIs), adoptive T-cell therapies, tumor vaccines, and oncolytic viral (OV) therapies (Fig. 1 and Tables 1, 2).

**Table 1:** Completed clinical trials of immunotherapy alone and combinations in GBM

Treatment	Target	Trial Number	Phase	Patient population	Design	Results	References
**Immune checkpoint inhibitors**							
Nivolumab	PD-1	NCT02617589	III	Newly diagnosed GBM, MGMT unmethylated	Nivolumab+RT vs. TMZ + RT	mOS 13.4 vs. 14.9 months; mPFS 6.0 vs. 6.2 months	[29]
Nivolumab	PD-1	NCT02667587	III	Newly diagnosed GBM, MGMT methylated	Nivolumab+RT + TMZ vs. placeo+RT + TMZ	mOS 28.9 vs. 32.1 months; mPFS 10.6 vs. 10.3 months	[30]
Nivolumab	PD-1	NCT02017717	III	Recurrent GBM	Nivolumab vs. bevacizumab	mOS 9.8 vs. 10.0 months; mPFS 1.5 vs. 3.5 months; ORR 7.8 vs. 23.1%	[31]
Pembrolizumab	PD-1	NCT02054806	Ib	PD-L1^+^ Recurrent GBM	Pembrolizumab	mOS 13.1 months; mPFS 2.8 months; ORR 8%	[32]
Pembrolizumab	PD-1	NCT02337491	II	Recurrent GBM	Pembrolizumab+bevacizumab vs. pembrolizumab	mOS 8.8 vs. 10.3 months; 6-month PFS rate 26.0 vs. 6.7%; ORR 20 vs. 0%	[33]
Pembrolizumab	PD-1	NCT02337686	II	Recurrent GBM	Pembrolizumab	mOS 20.0 months; mPFS 4.5 months	[34]
Pembrolizumab	PD-1	N/A	II	Recurrent GBM	Neoadjuvant pembrolizumab vs. adjuvant pembrolizumab	mOS 13.7 vs. 7.5 months; mPFS 3.3 vs. 2.4 months	[36]
Avelumab	PD-L1	NCT03291314	II	Recurrent GBM	Avelumab+axitinib	mOS 6.2 months; 6-month PFS 22.2%	[38]
Durvalumab	PD-L1	NCT02336165	II	Newly diagnosed GBM, MGMT unmethylated	Durvalumab+RT	mOS 15.1 months	[39]
Durvalumab	PD-L1	NCT02336165	II	Newly diagnosed and recurrent GBM	Newly diagnosed GBM: Durvalumab+RT (A); Bevacizumab-naïve, recurrent GBM: Durvalumab alone (B1), Durvalumab+bevacizumab (B2), Durvalumab+low-dose bevacizumab (B3); Bevacizumab-refractory, recurrent GBM: Durvalumab+bevacizumab (C)	mOS 15.1 (A), 6.7 (B1), 8.7 (B2), 9.3 (B3), 4.5 (C) months; mPFS 4.6 (A), 3.0 (B1), 3.7 (B2), 3.7 (B3), 1.9 (C) months	[40]
Atezolizumab	PD-L1	NCT01375842	Ia	Recurrent GBM	Atezolizumab	mOS 4.2 months	[41]
Ipilimumab	CTLA-4	ISRCTN84434175	II	Newly diagnosed GBM	Ipilimumab+TMZ vs. TMZ	mOS 22.7 vs. 26.4 months; mPFS 10.9 vs. 12.5 months	[42]
Ipilimumab+nivolumab	CTLA-4, PD-1	NCT02017717	I	Recurrent GBM	Ipilimumab+nivolumab vs. nivolumab	mOS 9.2 vs. 10.4 months; mPFS 1.5 vs. 1.9 months	[43]
Ipilimumab+nivolumab	CTLA-4, PD-1	NCT03233152	I	Recurrent GBM	Ipilimumab+nivolumab	mOS 8.9 months; mPFS 2.7 months	[44]
**Adoptive T cell therapies**							
CMV-specific T cells	CMV	ACTRN12615000656538	I	Primary GBM	CMV-specific T cells	mOS 23.0 months for patients treated before recurrence	[53]
CAR-T cells	IL-13Rα2	NCT02208362	I	Recurrent GBM	ICT, ICV, dual ICT/ICV administration	mOS 10.2 months for patients treated with dual ICT/ICV	[61]
CAR-T cells	IL-13Rα2	NCT01082926	I	GBM	IL-13Rα2-targeted CAR-T cells+IL-2	Transient tumor reduction or necrosis	[63]
CAR-T cells	EGFRvIII	NCT02209376	I	EGFRvIII^+^ recurrent GBM	EGFRvIII-targeted CAR-T cells	mOS 8.4 months	[66]
CAR-T cells	EGFRvIII	NCT05660369	I	Newly diagnosed and recurrent GBM	CARv3-TEAM-E T cells	Dramatic radiographic tumor regression	[67]
CAR-T cells	IL-13Rα2, EGFR	NCT05168423	I	Recurrent GBM	CAR-EGFR-IL13Rα2 T cells	Tumor reduction in all patients but none met the criteria for ORR	[68]
**Cancer vaccines**							
CDX-110	EGFRvIII	NCT01480479	III	Newly diagnosed, EGFRvIII^+^ GBM	CDX-110 + TMZ vs. TMZ	mOS 20.1 vs. 20.0 months; mPFS 8.0 vs. 7.4 months	[80]
SurVaxM	survivin	NCT02455557	II	Newly diagnosed GBM	SurVaxM+TMZ	mOS 25.9 months; mPFS 11.4 months	[83]
IMA950	11 TAAs	NCT01222221	I	Newly diagnosed GBM	IMA950 + GM-CSF	mOS 15.3 months	[85]
IMA950	11 TAAs	NCT01920191	I/II	Newly diagnosed GBM	IMA950+poly-ICLC	mOS 19 months	[88]
HSPPC-96	TSAs	NCT00293423	II	Recurrent GBM	HSPPC-96 vaccine after gross total resection	mOS 9.9 months	[92]
Personalized vaccine	TSAs	NCT02149225	I	Newly diagnosed GBM	Personalized vaccines+GM-CSF+poly-ICLC	mOS 29.0 months; mPFS 14.2 months	[95]
Personalized vaccine	TSAs	NCT02287428	Ib	Newly diagnosed GBM, MGMT unmethylated	Personalized vaccines+poly-ICLC	mOS 16.8 months; mPFS 7.6 months	[96]
Personalized vaccine	TSAs	N/A	III	Recurrent GBM	Personalized vaccines vs. placeo	mOS 8.4 vs. 8.0 months	[97]
DCVax-L	Autologous tumor antigens	NCT00045968	III	Newly diagnosed and recurrent GBM	DCVax-L vs. placebo	mOS 23.1 months	[100]
ICT-107	TAAs	NCT01280552	II	Newly diagnosed GBM	ICT-107 + SOC vs. placebo +SOC	mOS 17.0 vs. 15.0 months; mPFS 11.2 vs. 9.0 months	[102]
CMV-DC	CMV-pp65	NCT00639639	II	Newly diagnosed GBM	CMV DC + TMZ + GM-CSF	mOS 41.1 months; mPFS 25.3 months	[104]
VXM01	VEGFR-2	NCT02718443	I	Recurrent GBM	VXM01	3 patients with PR, 3 patients with SD	[110]
VBI-1901	CMV antigens (gB and pp65)	NCT03382977	IIa	Recurrent GBM	VBI-1901 + GM-CSF	mOS 12.9 months; 12-month OS rate 62.5%	[115]
**Oncolytic viral therapies**							
G207	HSV targeting tumor cells	NCT00157703	I	Recurrent GBM	G207 + RT	mOS 7.5 months; mPFS 2.5 months	[127]
G47∆	HSV targeting tumor cells	UMIN000002661	I/II	Recurrent GBM	G47∆ + RT + TMZ	mOS 7.3 months	[130]
G47∆	HSV targeting tumor cells	UMIN000015995	II	Recurrent GBM	G47∆ + RT + TMZ	mOS 20.2 months	[131]
PVSRIPO	Poliovirus targeting tumor cells	NCT01491893	I	Recurrent GBM	PVSRIPO vs. historical controls	mOS 12.5 vs. 11.3 months; 24-month OS rate 21 vs. 14%; 36-month OS rate 21 vs. 4%	[134]
DNX-2401	Adenovirus targeting tumor cells	N/A	I	Recurrent GBM	DNX-2401	mOS 4.6 months; mPFS 2.7 months	[136]
DNX-2401	Adenovirus targeting tumor cells	NCT02197169	Ib	Recurrent GBM	DNX-2401 + IFN-γ vs. DNX-2401	Poor tolerability	[139]
CRAd-S-pk7	Adenovirus targeting tumor cells	NCT03072134	I	Newly diagnosed GBM	NSC-CRAd-S-pk7	mOS 18.4 months; mPFS 9.1 months	[143]
Ad-RTS-hIL-12	Adenovirus delivering IL-12	NCT02026271	I	Resected recurrent GBM	Ad-RTS-hIL-12	mOS 12.7 months with 20 mg VDX	[146]
VB-111	Adenovirus delivering Fas-chimera transgene	NCT01260506	I/II	Recurrent GBM	VB-111+bevacizumab vs. VB-111	mOS 13.8 vs. 7.4 months; mPFS 3.0 vs. 3.0 months	[148]
VB-111	Adenovirus delivering Fas-chimera transgene	NCT02511405	III	Recurrent GBM	VB-111+bevacizumab vs. bevacizumab	mOS 6.8 vs. 7.9 months; mPFS 3.4 vs. 3.7 months	[149]
AdV-tk	Adenovirus delivering HSV-TK	NCT00870181	II	Recurrent GBM	AdV-tk vs. conventional treatment	mOS 10.6 vs. 2.0 months; mPFS 8.1vs. 1.7 months	[153]
AdV-tk	Adenovirus delivering HSV-TK	NCT00589875	II	Newly diagnosed GBM	AdV-tk+SOC vs. SOC	mOS 25.1 vs. 16.3 months	[154]
AdV-tk	Adenovirus delivering HSV-TK	N/A	III	Newly diagnosed and recurrent GBM	AdV-tk+SOC vs. SOC	mOS 12.9 vs. 8.6 months	[155]
AdV-tk	Adenovirus delivering HSV-TK	EudraCT2004-000464-28	III	Newly diagnosed GBM	AdV-tk+SOC vs. SOC	mOS 16.6 vs. 15.1 months; mPFS 10.3 vs. 8.9 months	[156]
AdV-tk	Adenovirus delivering HSV-TK	NCT01811992	I	Newly diagnosed GBM	AdV-tk+AdV-Flt3L	mOS 21.3 months	[157]
Retrovirus	Retrovirus delivering HSV-TK	N/A	III	Newly diagnosed GBM	HSV-TK + SOC vs. SOC	mOS 12.0 vs. 11.8 months; mPFS 6.0 vs. 6.1 months	[158]
Toca 511/FC	Retrovirus targeting tumor cells	NCT02414165	II/II	Recurrent GBM	Toca 511/FC vs. SOC	mOS 11.1 vs. 12.2 months; mPFS 6.0 vs. 6.1 months	[162]

*CAR* chimeric antigen receptor, *CAR VSTs* CAR-modified virus-specific T cells, *CTLA-4* cytotoxic T-lymphocyte associated protein 4, *CMV* cytomegalovirus, *EGFRvIII* epidermal growth factor receptor variant III, *Flt3L* FMS-like tyrosine kinase 3 ligand, *GBM* glioblastoma, *GM-CSF* granulocyte-macrophage colony-stimulating factor, *HER2* human epidermal growth factor receptor 2, *HSPPC-96* heat shock protein peptide complexes 96, *HSV* herpes simplex virus, *HSV-TK* herpes simplex virus thymidine kinase, *ICT* intratumoral, *ICV* intraventricular, *hIL-2* human interleukin-2, *IL-13Rα2* interleukin-13 receptor subunit alpha 2, *IL-2* interleukin-2, *MGMT* O^6^-methylguanine-DNA methyltransferase, *mOS* median overall survival, *mPFS* median progression-free survival, *ORR* objective response rate, *PD-1* programmed cell death protein 1, *PD-L1* programmed cell death ligand 1, *Poly-ICLC* polyinosinic-polycytidylic acid with polylysine and carboxymethylcellulose, *RT* radiotherapy, *SOC* standard of care, *TAAs* tumor-associated antigens, *TEAM* T cell-engaging antibody molecule, *TMZ* temozolomide, *TSAs* tumor-specific antigens

**Table 2:** Ongoing clinical trials of immunotherapy alone and combinations in GBM

Treatment	Target	NCT Number	Phase	Patient population	Design
**Immune checkpoint inhibitors**					
Nivolumab, ipilimumab	PD-1, CTLA-4	NCT04396860	II/III	Newly diagnosed GBM, MGMT unmethylated	RT + TMZ vs. RT+nivolumab+ipilimumab
Nivolumab, BMS-986016	PD-1, LAG-3	NCT03493932	I	Recurrent GBM	Nivolumab+BMS-986016
BMS-986016, urelumab, nivolumab	LAG-3, CD137, PD-1	NCT02658981	I	Recurrent GBM	BMS-986016 (A1), Urelumab (A2), BMS-986016+Nivolumab (B1), Urelumab+Nivolumab (B2)
MBG453, spartalizumab	TIM-3, PD-1	NCT03961971	I	Recurrent GBM	Stereotactic radiosurgery+MBG453+spartalizumab
AB154, AB122	TIGIT, PD-1	NCT04656535	0/I	Recurrent GBM	AB154 + AB122 (Safety A), AB154 (B1), AB122 (B2), AB154 + AB122 (B3), Placebo (B4)
BMS-986205, nivolumab	IDO1, PD-1	NCT04047706	I	Newly diagnosed GBM	BMS-986205+Nivolumab+RT + TMZ vs. BMS-986205+Nivolumab+RT
Indoximod, TMZ, bevacizumab	IDO1	NCT02052648	I/II	Recurrent GBM	Indoximod+TMZ (I), Indoximod+TMZ+bevacizumab (II), Indoximod+TMZ + RT (III)
**Adoptive T cell therapies**					
TILs	Tumor cell	NCT05333588	I	Advanced Stage of GBM	TILs
TILs	Tumor cell	NCT04943913	I	Malignant glioma	TILs
CAR-T cells	PD-1	NCT02873390	I/II	EGFR^+^ Advanced Solid Tumor	Herin CAR-PD-1 cells
CAR-T cells	PD-L1	NCT02937844	I	Recurrent GBM	Anti-PD-L1 CAR T cells
CAR-T cells	CTLA-4, PD-1	NCT03182816	I/II	EGFR^+^ Advanced Solid Tumor	Anti-CTLA-4/PD-1 expressing EGFR-CAR-T cells
CAR-T cells	IL-13Rα2	NCT04003649	I	Recurrent GBM	IL-13Rα2-CAR T cells+nivolumab+ipilimumab (I), IL-13Rα2-CAR T cells+nivolumab (II), IL-13Rα2-CAR T cells (III)
**Cancer vaccines**					
INO-5401, INO-9012	hTERT, WT-1, PSMA	NCT03491683	I/II	Newly diagnosed GBM	INO-5401 + INO-9012+cemiplimab+RT + TMZ (MGMT unmethylated vs. MGMT methylated)
SurVaxM	Survivin	NCT02455557	II	Newly diagnosed GBM	SurVaxM+TMZ
IMA950	11 TAAs	NCT03665545	I/II	Recurrent GBM	IMA950+Poly-ICLC vs. IMA950/Poly-ICLC+pembrolizumab
Personalized vaccine	TSAs	NCT05743595	I	Newly diagnosed GBM, MGMT unmethylated	Personalized neoantigen DNA vaccine+retifanlimab
ATL-DCs	Autologous tumor antigens	NCT04201873	I	Recurrent GBM	Pembrolizumab+ATL-DCs+poly-ICLC vs. Placebo+ATL-DCs+poly-ICLC
**Oncolytic viral therapies**					
PVSRIPO	Poliovirus targeting tumor cells	NCT04479241	II	Recurrent GBM	PVSRIPO+pembrolizumab
PVSRIPO	Poliovirus targeting tumor cells	NCT02986178	II	Recurrent GBM	PVSRIPO
PVSRIPO	Poliovirus targeting tumor cells	NCT03973879	I/II	Recurrent GBM	PVSRIPO+atezolizumab
PVSRIPO	Poliovirus targeting tumor cells	NCT04479241	II	Recurrent GBM	PVSRIPO+pembrolizumab
PVSRIPO	Poliovirus targeting tumor cells	NCT03973879	I/II	Recurrent GBM	PVSRIPO+atezolizumab
AdV-tk	Adenovirus delivering HSV-TK	NCT03576612	I	Newly diagnosed GBM	AdV-tk+nivolumab+RT + TMZ (MGMT unmethylated vs. MGMT methylated)
Ad-RTS-hIL-12	Adenovirus targeting tumor cells	NCT04006119	II	Recurrent or progressive GBM	Ad-RTS-hIL-12+cemiplimab

*ATL-DCs* autologous tumor lysate pulsed dendritic cells, *CAR* chimeric antigen receptor, *CTLA-4* cytotoxic T-lymphocyte associated protein 4, *EGFR* epidermal growth factor receptor, *GBM* glioblastoma, *HSV-TK* herpes simplex virus thymidine kinase, *hTERT* human telomerase reverse transcriptase, *IL-13Ra2* interleukin-13 receptor subunit alpha 2, *LAG-3* lymphocyte activation gene 3, *MGMT* O^6^-methylguanine-DNA methyltransferase, *PD-1* programmed cell death protein 1, *PD-L1* programmed cell death ligand 1, *Poly-ICLC* polyinosinic-polycytidylic acid with polylysine and carboxymethylcellulose, *PSMA* prostate-specific membrane antigen, *RT* radiotherapy, *TAAs* tumor-associated antigens, *TIGIT* T cell immunoreceptor with immunoglobulin and ITIM domain, *TILs* tumor infiltrating lymphocytes, *TIM-3* T cell immunoglobulin and mucin domain 3, *TMZ* temozolomide, *WT-1* wilms’ tumor 1

### Immune checkpoint inhibitors

Immune checkpoints are a class of regulatory molecules expressed on the surface of certain immune cells, particularly T cells, that are crucial for maintaining immune system homeostasis and preventing autoimmunity [14–16]. Under physiological conditions, these molecules are essential for self-tolerance and protecting tissues from immune-related damage [17–20]. However, in the context of cancer, they can be exploited by tumor cells to evade immune surveillance and suppress antitumor immunity [15, 16, 21, 22]. In the past few decades, several immune checkpoints (Fig. 1A) have been identified, including programmed cell death protein 1 (PD-1) and its ligand PD-L1, cytotoxic T-lymphocyte associated protein 4 (CTLA-4), lymphocyte activation gene 3 (LAG-3), T-cell immunoreceptor with immunoglobulin and ITIM domain (TIGIT), T-cell immunoglobulin and mucin domain 3 (TIM-3), V-domain Ig suppressor of T-cell activation (VISTA), and indoleamine 2,3-dioxygenase 1 (IDO1) [14, 23–26]. Blocking these immune checkpoints with monoclonal antibodies, known as ICIs, has shown significant success in treating multiple advanced cancers, such as melanoma, non-small cell lung cancer, colorectal cancer, gastric cancer, and hepatocellular carcinoma [10, 27]. However, a large number of clinical trials have shown minimal clinical benefit for GBM patients [28].

Three phase III trials have been designed to test the antitumor efficacy of the anti-PD-1 antibody nivolumab in GBM patients [29–31]. In the CheckMate 498 study, 560 newly diagnosed GBM patients with an unmethylated O^6^-methylguanine-DNA methyltransferase (MGMT) promoter received RT in combination with nivolumab or TMZ. However, the study did not meet its primary endpoint of OS, with mOS values of 13.4 and 14.9 months for the nivolumab and TMZ groups, respectively [29]. Similarly, the CheckMate 548 study involving 716 newly diagnosed GBM patients with a methylated MGMT promoter also failed to improve the mOS (28.9 vs. 32.1 months) or median progression-free survival (mPFS, 10.6 vs. 10.3 months) upon the addition of nivolumab concurrent with the SOC [30]. In another setting, the CheckMate 143 study compared the antitumor efficacy of nivolumab combined with the antiangiogenic drug bevacizumab in recurrent GBM patients. This study enrolled 369 GBM patients at their first recurrence following RT and TMZ therapy and revealed that nivolumab treatment did not improve patients’ mOS (9.8 vs. 10.0 months), mPFS (1.5 vs. 3.5 months), or objective response rate (ORR, 7.8 vs. 23.1%) [31]. Similarly, another anti-PD-1 antibody, pembrolizumab, has shown limited clinical benefit both as monotherapy and in combination with bevacizumab for recurrent GBM patients in phase I and II trials [32–34]. Notably, neoadjuvant anti-PD-1 immunotherapy has demonstrated encouraging outcomes in selected recurrent GBM patients in window-of-opportunity trials [35, 36]. For example, a notable study with 35 recurrent GBM patients demonstrated that neoadjuvant pembrolizumab therapy combined with adjuvant therapy prolonged patient survival compared with patients who received only postsurgical pembrolizumab treatment (mOS, 13.7 vs. 7.5 months; mPFS, 3.3 vs. 2.4 months) [36]. Mechanistic studies have demonstrated that this neoadjuvant pembrolizumab therapy increases T-cell infiltration and interferon-γ (IFN-γ)-related gene expression [36] and enhances chemokine expression and T-cell receptor (TCR) clonal diversity [37]. In addition to these investigations using anti-PD-1 antibodies, the efficacy and safety of PD-L1 antibodies (e.g., durvalumab, atezolizumab, and avelumab) have also been evaluated in GBM patients, with results showing potential antitumor efficacy and favorable tolerability [38–41].

For the anti-CTLA-4 antibody ipilimumab, a phase II clinical trial has been performed, and the results showed that ipilimumab combined with TMZ treatment did not lead to clinical benefit over TMZ treatment alone (mOS, 22.7 vs. 26.4 months; mPFS, 10.9 vs. 12.5 months) [42]. Another ongoing randomized open-label phase II/III trial (NCT04396860) aims to test the antitumor effect of combining ipilimumab, nivolumab, and RT in newly diagnosed and unmethylated MGMT GBM patients. For recurrent GBM patients, the phase I CheckMate 143 trial revealed that intravenous administration of ipilimumab and nivolumab has no clinical benefit but increases immune toxicity compared with nivolumab treatment alone (mOS, 9.2 vs. 10.4 months; mPFS, 1.5 vs. 1.9 months) [43]. However, one phase I clinical trial in recurrent GBM patients involving the intracerebral administration of ipilimumab and nivolumab showed a promising survival benefit (mOS, 8.9 months; mPFS, 2.7 months), encouraging further investigations [44].

In addition to “classic” immune checkpoints, novel targets such as LAG-3, TIM-3, TIGIT, and IDO1 are actively under investigation for GBM treatment. Given that LAG-3 is an early marker of exhausted T cells, treatment with anti-LAG-3 drugs might offer therapeutic benefits in cancer patients [45]. In the context of GBM, two phase I clinical trials (NCT02658981 and NCT03493932) are underway to explore the antitumor effect of the LAG-3 inhibitor relatlimab (BMS-986016) alone or in combination with nivolumab in recurrent or newly diagnosed GBM patients. TIM-3 is a coinhibitory molecule expressed on immune cells, and TIM-3-targeted therapies are currently under investigation in various cancer types, including GBM [46]. For example, treatment with the TIM-3 inhibitor sabatolimab combined with the PD-1 inhibitor spartalizumab and stereotactic radiosurgery is underway for recurrent GBM patients (NCT03961971). TIGIT is a T-cell coinhibitory receptor that is expressed mainly by T cells and natural killer (NK) cells [47]. Preclinical studies have shown that inhibition of TIGIT improves the antitumor immune response [47]. A phase 0/I study (NCT04656535) is recruiting recurrent GBM patients to evaluate the safety and tolerability of the anti-TIGIT antibody domvanalimab (AB154) combined with the anti-PD-1 antibody zimberelimab (AB122). In addition, the antitumor efficiency of IDO1 inhibitors (e.g., BMS-986205 and indoximod) combined with RT and TMZ is also currently under investigation in different clinical trials (NCT04047706 and NCT02052648) for newly diagnosed GBM patients.

### T-cell therapies

Adoptive T-cell therapy is a personalized cancer immunotherapy technique in which a patient’s T cells are isolated, expanded ex vivo and then reinfused back into the patient to target tumors [48]. Before reinfusion, patients undergo a lymphodepleting regimen with lymphocyte-directed chemotherapy to increase treatment effectiveness by creating a favorable environment for infused cells [48]. An increasing number of clinical trials have demonstrated a transient antitumor response with manageable side effects, providing hope for the development of effective adoptive T-cell therapies for GBM. Here, we discuss the current status of adoptive T-cell therapies, including tumor-infiltrating lymphocyte (TIL) therapy, cytomegalovirus (CMV)-specific T-cell therapy, and chimeric antigen receptor (CAR)-T-cell therapy, in GBM (Fig. 1B).

#### Tumor-infiltrating lymphocyte therapy

The autologous TILs for adoptive T-cell therapy are prepared by culturing a resected tumor specimen in a high concentration of recombinant interleukin-2 (IL-2), along with IL-15 and IL-21 if necessary, then selecting and expanding them in vitro and transferring them back to the patient [49]. This is a time-consuming process with a low success rate, and its application is limited. A pilot study revealed that autologous TILs combined with IL-2 had limited antitumor effects on malignant gliomas, potentially due to the heterogeneity of TILs in terms of TCR diversity and exhaustion levels [50]. Two other phase I clinical trials (NCT05333588 and NCT04943913) are currently recruiting GBM patients to evaluate the safety of TIL therapy. In addition, engineered autologous TILs that secrete antibodies targeting PD-1 (PD-1-TILs, aiming to prevent infused T-cell exhaustion induced by PD-1-mediated immunosuppression) are well tolerated, with no unexpected high-grade adverse events in GBM patients, and show improved antitumor efficacy compared with that of normal TILs, representing a new TIL treatment strategy for GBM [51].

#### Cytomegalovirus-specific T-cell therapy

Autologous CMV-specific T-cell therapy is another feasible adoptive T-cell therapy since the majority of GBM tumors, but not the surrounding normal brain tissues, express CMV antigens, which contribute to GBM progression [52]. To this end, peripheral blood mononuclear cells are isolated from the blood and expanded in vitro with synthetic CMV peptides to generate CMV-specific T cells, which are then reinfused back into the patient [52]. A phase I study revealed that autologous CMV-specific T cells are safe for primary GBM patients and provide encouraging clinical evidence for improving patient survival if therapy is offered before tumor recurrence (mOS, 23.0 vs. 14.0 months) [53]. However, larger controlled trials are needed to reproduce these observations.

#### Chimeric antigen receptor-T-cell therapy

A significant advancement in adoptive cell therapy is the development of CAR-T-cell therapy [54]. The autologous T cells can be genetically engineered to express CARs, which combine the antigen recognition domains of antibodies with T-cell activation domains derived from the CD3ζ chain and costimulatory receptors, such as CD28 and 4-1BB [55]. This enables CAR-T cells to specifically target tumor antigens in an MHC-independent manner, thereby bypassing antigen presentation and enhancing their ability to recognize and kill cancer cells [55]. Despite the remarkable clinical response to CAR-T-cell therapy in patients with hematologic malignancies, such as acute lymphoblastic leukemia [56] and diffuse large B-cell lymphoma [57], extending this strategy to solid tumors (e.g., GBM) is still challenging. However, significant work has aimed to develop CAR-T-cell therapies for GBM. Here, we discuss several tumor antigens, such as interleukin-13 receptor subunit alpha 2 (IL-13Rα2) and epidermal growth factor receptor variant III (EGFRvIII), that have been targeted to develop CAR-T-cell therapies for GBM treatment.

##### IL-13Rα2

Initial efforts in CAR-T-cell therapy focused on IL-13Rα2 since it is expressed in more than 75% of GBM cases [58]. A pilot study evaluated the safety and feasibility of CD8^+^ CAR-T cells targeting IL-13Rα2 in three recurrent GBM patients, and the results revealed a good safety profile and transient antitumor activity [59]. Another investigation of a recurrent GBM patient used dual-route CAR-T-cell infusions, including intracranial infusion into the resection cavity followed by intrathecal delivery into the ventricular system, which led to a favorable clinical response with the regression of all intracranial and spinal tumors for a duration of 7.5 months without severe toxicity [60]. Following these two breakthrough studies, Brown et al. conducted a phase I clinical trial in 65 patients with recurrent high-grade glioma (the majority of whom had recurrent GBM) by administering IL-13Rα2-targeted CAR-T cells through three different routes, including intratumoral (ICT), intraventricular (ICV), and dual ICT/ICV. This study confirmed the feasibility and safety of locoregional CAR-T-cell administration. Stable disease or better clinical outcomes were observed in 50% of patients. The mOS for recurrent GBM patients increased to 10.2 months from 7.7 months after dual ICT/ICV IL-13Rα2-targeted CAR-T-cell treatment [61]. Moreover, another phase I trial explored the feasibility and safety of off-the-shelf allogeneic IL-13Rα2-targeted CAR-T cells that were genetically engineered to resist treatment with glucocorticoids, which are commonly prescribed to control cerebral edema and inflammation in GBM, in six patients with unresectable recurrent GBM [62]. The results revealed that local intracranial administration of IL-13Rα2-targeted CAR-T-cell therapy in combination with recombinant human IL-2 was well tolerated and resulted in transient tumor reduction or necrosis at the infusion site in four patients [63]. Although all of these patients experienced GBM recurrence during the study, these findings provide a new direction for adoptive therapy using off-the-shelf, zinc finger-modified, and/or glucocorticoid-resistant CAR-T cells. Together, these findings highlight the potential of IL-13Rα2-targeted CAR-T-cell therapy for recurrent GBM, and further investigations are merited.

##### EGFRvIII

EGFRvIII is a mutated variant of the EGFR with deletion of exons 2-7 of the *EGFR* gene [64]. EGFRvIII is a tumor-specific protein in GBM that represents the predominant form of EGFR in tumors, with approximately half of the EGFR-amplified GBM cases expressing this variant [65]. Despite the accumulation of promising preclinical data, the clinical efficacy of CAR-T cells targeting EGFRvIII in GBM patients remains limited. In a first-in-human phase I clinical trial, ten patients with recurrent EGFRvIII^+^ GBM received a single intravenous infusion of autologous EGFRvIII-directed CAR-T cells. The treatment was proven to be safe and feasible (without cross-reactivity to wild-type EGFR, cytokine-release syndrome, or neurotoxicity) and was shown to downregulate tumoral EGFRvIII and upregulate immunosuppressive factors (e.g., IDO1 and PD-L1) and regulatory T cells (Tregs). However, the mOS of these patients was not affected by this therapy [66]. The treatment selective pressure induced by EGFRvIII antigen escape might contribute to the low efficacy of EGFRvIII-directed CAR-T-cell therapy in GBM patients. To address this issue, an engineered T-cell product (named CARv3-TEAM-E) has been developed to target EGFRvIII through a second-generation CAR and secrete a T-cell-engaging antibody molecule (TEAM, a bispecific antibody) that can recognize wild-type EGFR in GBM cells and CD3 in T cells. The intraventricular administration of CARv3-TEAM-E resulted in a promising safety profile and dramatic radiographic tumor regression. However, it should be noted that the antitumor responses were transient in two out of three patients [67]. Given the heterogeneous expression of target antigens in GBM cells, targeting multiple antigens could be a possible direction for developing new CAR-T-cell therapies. Indeed, a recent phase I clinical trial in six patients with multifocal recurrent GBM showed that the intrathecal injection of bivalent CAR-T cells targeting both IL-13Rα2 and EGFR led to tumor reduction in all patients, although none met the criteria for ORR [68]. Overall, these first-in-human data prove the efficacy and safety of EGFR-IL13Rα2-targeted CAR-T cells in recurrent GBM.

### Cancer vaccines

Cancer vaccines aim to activate the patient’s adaptive immune system against specific endogenous and exogenous tumor antigens and are being explored in GBM [69]. Endogenous antigens, including tumor-specific antigens (TSAs, also known as neoantigens) and tumor-associated antigens (TAAs), are intracellular proteins from tumor cells. Exogenous antigens originate from the infection of exogenous pathogens, such as CMV [70, 71]. Cancer vaccines can be created using “predefined” antigens (known antigens, which include shared antigens expressed in the majority of patient tumors or personalized antigens specific to individual patients) or “anonymous” antigens (unknown antigens, which can be colocalized with antigen-presenting cells (APCs) ex vivo or engineered for reinjection into patients to activate endogenous APCs in situ) [72, 73]. After administration, antigens are presented by APCs to naïve or memory T cells, and these primed T cells then migrate into tumor sites, initiating tumor regression and establishing long-lasting memory responses against tumor recurrence [72, 73]. Various vaccination strategies are under investigation for GBM treatment [69]. In this section, we discuss four different vaccine platforms, including peptide vaccines, dendritic cell (DC) vaccines, DNA/RNA vaccines, and viral vector vaccines, and their use for GBM treatment (Fig. 1C).

#### Peptide vaccines

Peptide vaccines utilize synthetic peptides to mimic TSA or TAA epitopes, producing new or enhanced tumor-specific T-cell responses [74]. These peptides can be engulfed and presented by APCs to autologous CD4^+^ and CD8^+^ T cells after in vivo administration and then circulate to the tumor site, where they exhibit cytotoxicity [74]. Given the inability of these peptides to activate the innate immune system, additional immune adjuvants are usually combined with peptide vaccines, ensuring sufficient costimulatory signals from APCs to elicit a robust T-cell response [75]. Here, we discuss the common peptide vaccines used in GBM, which include rindopepimut (CDX-110), survivin vaccine (SurVaxM), IMA950, heat shock protein peptide complex-96 (HSPPC-96)-specific vaccine, and personalized neoantigen vaccines.

##### CDX-110

CDX-110 is a 13-amino acid peptide vaccine targeting the EGFRvIII mutation, and it consists of an EGFRvIII-specific peptide coupled with keyhole limpet hemocyanin to increase immunogenicity. Despite the promising results from phase II clinical trials [76–79], the phase III trial (NCT01480479) with CDX-110 treatment combined with chemotherapy failed to show clinical benefits in newly diagnosed GBM patients compared with chemotherapy alone (mOS, 20.1 vs. 20.0 months; mPFS, 8.0 vs. 7.4 months) [80]. Notably, approximately 60% of the patients (regardless of receiving CDX-110 treatment) experienced EGFRvIII loss, underscoring the necessity for biopsy-confirmed EGFRvIII expression before enrollment for treatment [80]. In addition, the dynamic nature of EGFRvIII raises concerns about its reliability as a stable target for vaccine development [64], suggesting the necessity of developing novel therapeutic strategies with multitarget potential.

##### SurVaxM

The SurVaxM vaccine targets survivin, an antiapoptotic protein that is highly expressed in GBM tumors but not in normal brain tissues [81]. An early phase I clinical trial (NCT01250470) evaluated its safety in 9 survivin^+^ recurrent glioma patients [82]. Moreover, a recent phase II study (NCT02455557) in newly diagnosed GBM patients demonstrated that SurVaxM treatment combined with TMZ resulted in improved mPFS (11.4 months) and mOS (25.9 months) compared with those of historical controls [83]. A multicenter and randomized controlled phase IIb trial (SURVIVE) is underway to investigate the efficacy and safety of SurVaxM combined with adjuvant TMZ in newly diagnosed GBM patients [84].

##### IMA950

IMA950 is a multipeptide vaccine consisting of 11 TAAs (9 MHC class I-restricted peptides and 2 MHC class II-restricted peptides) that are overexpressed in GBM cells. The first human phase I trial with IMA950 in newly diagnosed GBM (NCT01222221) demonstrated that treatment with IMA950 in combination with granulocyte‒macrophage colony‒stimulating factor (GM-CSF) was well tolerated and generated potent T-cell immune responses in at least 30% of patients [85]. Similarly, a phase I/II trial (NCT01920191) in 16 newly diagnosed GBM patients revealed good tolerability and immunogenicity when IMA950 was combined with the adjuvant immunostimulant poly-ICLC, a synthetic analog of dsRNA and toll-like receptor 3 ligand [86, 87]. Notably, the mOS of these patients reached 19.0 months, although 4 patients experienced short-term cerebral edema [88]. Although the IMA950/poly-ICLC peptide vaccine has shown no clinical benefit for recurrent GBM [89], a phase II clinical trial (NCT03665545) was designed to evaluate the antitumor efficacy of the IMA950/poly-ICLC vaccine combined with the anti-PD-1 antibody pembrolizumab in recurrent GBM patients [90].

##### HSPPC-96

Heat shock proteins (HSPs) are overexpressed in many cancers, where they contribute to cancer cell proliferation, differentiation, infiltration, and metastasis [91]. HSPs and autologous tumor antigen polypeptides can form complexes, named HSP-peptide complexes (HSPPCs), to mediate cell endocytosis and antigen presentation by binding to APC membrane receptors, activating CD4^+^ and CD8^+^ T cells and triggering immune responses against tumor antigen peptides [92]. The HSPPC-96 vaccine consists of the HSP glycoprotein 96 and its associated cellular neopeptides, which target multiple TSAs [93]. A phase II study evaluated the safety and antitumor efficacy of the HSPPC-96 vaccine in recurrent GBM patients and reported an mOS of 9.9 months [92]. Further studies demonstrated that the HSPPC-96 vaccine can improve the survival of GBM patients who undergo SOC and revealed that peripheral myeloid cell expression of PD-L1 might impact the antitumor efficacy of the vaccine [94].

##### Personalized neoantigen vaccines

Personalized polypeptide vaccines have been developed by utilizing whole-exome sequencing data to identify patient-specific neoantigens. These immune targets are designed individually according to the specific mutation site, type, and expression of the tumor neoantigen to reduce off-target effects and adverse effects. The European GAPVAC trial [95] and the American NEOVAX trial [96] have shown that these vaccines can stimulate circulating robust immune responses involving CD8^+^ and CD4^+^ T cells with a memory phenotype. The GAPVAC trial reported mPFS and mOS values of 14.2 and 29.0 months, respectively, in newly diagnosed GBM patients [95], whereas the NEOVAX trial reported mPFS and mOS values of 7.6 and 16.8 months, respectively, in newly diagnosed and unmethylated GBM patients [96]. However, another phase III trial for recurrent GBM did not meet its primary or secondary endpoints (personalized vaccine vs. placebo: mOS, 8.4 vs. 8.0 months) [97]. Thus, further studies are needed to validate and confirm the clinical benefit of personalized vaccines in GBM patients.

#### Dendritic cell vaccines

The intrinsic antigen presentation ability of DCs has stimulated researchers to create DC vaccine-based immunotherapies to activate adaptive immune responses [98, 99]. To make the vaccine, autologous DCs are harvested from the peripheral blood directly or differentiated from monocytes or CD34^+^ hematopoietic stem cells upon stimulation with cytokines, such as IL-4 and GM-CSF [98, 99]. These DCs are subsequently exposed to several forms of antigens, including DNA/RNA, peptides and tumor lysates. These tumor antigen-loaded DCs possess high antigen-presenting efficiency with sufficient exogenous costimulatory signals and can prime CD4^+^ T cells via peptide-MHC II complexes and CD8^+^ T cells via peptide-MHC I molecules [98, 99]. To date, several DC vaccines (e.g., DCVax-L, ICT-107, and CMV-DCs) have been tested in GBM patients.

##### DCVax-L

DCVax-L, an autologous DC vaccine pulsed with autologous tumor lysate ex vivo, is the most studied DC vaccine. The efficacy of DCVax-L was tested in a phase III clinical trial (NCT00045968) for newly diagnosed GBM patients after they received SOC. Patients were randomized at a 2:1 ratio to receive DCVax-L or placebo, with the option of crossover to the DCVax-L group in cases of disease progression or relapse during treatment [100]. Owing to the crossover design, approximately 90% of all GBM patients with recurrence received DCVax-L treatment, which resulted in depletion of the placebo group and necessitated the use of external controls for statistical analysis. Notably, compared with external controls, DCVax-L treatment led to increased mOS for both newly diagnosed GBM patients (19.3 vs. 16.5 months) and recurrent GBM patients (13.2 vs. 7.8 months) [101]. However, these survival data should be interpreted with caution considering the high crossover rate, a shift in the primary endpoint from PFS to OS, and an inappropriate selection of external controls.

##### ICT-107

ICT-107 is a DC vaccine consisting of autologous, monocyte-derived DCs pulsed with 6 well-known GBM TAAs, including melanoma-associated antigen 1, absent in melanoma 2, human epidermal growth factor receptor 2, tyrosinase-related protein 2, glycoprotein 100, and IL-13Rα2. A phase II study (NCT01280552) demonstrated the safety and immunogenicity of ICT-107 in newly diagnosed GBM patients. Although a modest improvement in PFS was observed in the ICT-107 group compared with the control group (11.2 vs. 9.0 months), the mOS was not significantly affected (17.0 vs. 15.0 months) [102].

##### CMV-DC vaccines

The CMV-DC vaccine (known as the CMV-pp65 RNA-pulsed DC vaccine) consists of autologous DCs pulsed with mRNA encoding the human CMV matrix protein pp65, which aims to kill GBM cells by stimulating CMV-specific T-cell immunity. Three separate clinical trials using CMV-DC vaccines have been conducted in newly diagnosed and SOC-treated GBM patients [103–105]. Notably, nearly one-third of GBM patients receiving CMV-DC vaccines exhibit no tumor recurrence at 5 years after diagnosis [105]. Given that CMV-DCs can trigger a CMV-specific CD8^+^ T-cell response [103, 104], they further conducted a pilot trial in which newly diagnosed GBM patients received both CMV pp65-specific T cells and a CMV-DC vaccine [106]. This combination treatment resulted in an increase in activated CMV-specific CD8^+^ T cells, which was correlated with better patient OS. However, further clinical trials should be conducted to confirm the antitumor effect of this treatment strategy in GBM patients.

#### DNA/RNA vaccines

Nucleic acid-based vaccines, including DNA (as plasmids) and RNA (as mRNA) vaccines, represent a new area of vaccine development for cancer treatment [107, 108]. DNA vaccines are generated by incorporating genes that encode TAAs into a bacteria-derived plasmid, which can induce robust CD4^+^ and CD8^+^ T-cell responses by presenting antigens on MHC class I/II molecules and producing humoral responses [107].

VXM01 is a DNA plasmid vaccine containing an attenuated strain of Salmonella typhimurium that encodes murine vascular endothelial growth factor receptor-2 (VEGFR-2), a protein that contributes to tumor angiogenesis [109]. A phase I clinical trial (NCT02718443) evaluated the antitumor efficiency of VXM01 in recurrent GBM patients who do not respond to SOC and revealed that VXM01 was well tolerated and elicited a VEGFR-2-specific T-cell immune response. Notably, a subset of patients with prolonged survival expresses lower intratumoral PD-L1, suggesting that the combination of VXM01 with anti-PD-L1 treatment is beneficial [110]. Another two DNA vaccines, INO-5401 (synthetic DNA plasmids encoding human telomerase reverse transcriptase, Wilms’ tumor gene 1, and prostate-specific membrane antigen), and INO-9012 (synthetic DNA plasmid encoding IL-12), in combination with the PD-1 inhibitor cemiplimab, are being investigated in an ongoing phase I/II trial (NCT03491683) for newly diagnosed GBM patients [111]. The safety, immunological effectiveness, and potential survival advantages of these treatments have been supported by interim results [112]. However, further trials are needed to validate the clinical benefits of these DNA plasmid vaccines in GBM patients.

mRNA vaccines transduce mRNA into cells, especially APCs, to generate translated peptides. This type of vaccine has significant safety advantages because it has the properties of rapid degradation and minimal risk of infection or insertional mutations [108]. To improve their preservation and membrane permeability, they are usually delivered by various packaging nanoparticles, such as lipid nanoparticles. One preclinical study demonstrated that mRNA-loaded lipid nanoparticles can induce a favorable antitumor response and sensitize poorly immunogenic murine GBM tumors to ICIs [113]. However, no clinical trials using mRNA vaccines to treat GBM have yet been reported.

#### Viral vector vaccines

Viral vector vaccines are novel vaccines that have been utilized for antigen delivery in clinical settings [114]. Genetically modified viral vectors can lose their toxicity and contain genes encoding targeted antigens, which, in turn, generate both innate and adaptive immune responses [114]. VBI-1901 is a viral vector vaccine that targets two highly immunogenic CMV antigens (glycoprotein B and phosphoprotein 65). The safety and antitumor efficacy of VBI-1901 in recurrent GBM patients have been evaluated in a randomized controlled phase IIa clinical trial (NCT03382977). Early data from this trial demonstrated that the mOS was 12.9 months, and the 12-month OS rate was 62.5% in 16 patients treated with the highest dose of VBI-1901 [115].

### Oncolytic viral therapies

OV therapy represents another class of immunotherapies being intensively investigated for GBM [116] (Fig. 1D). OVs are replication-competent viruses that selectively infect and replicate in cancer cells, causing lysis of GBM cells followed by the release of viral pathogen-associated molecular patterns, damage-associated molecular patterns, TAAs, and proinflammatory cytokines into the TME, which, in turn, induce antitumor immune responses and transform the TME from “cold” to “hot” [117]. Specifically, OVs can facilitate the activation and migration of APCs to activate cytotoxic CD8^+^ T cells, enhancing the overall immune response against GBM.

Herpes simplex virus (HSV) is the first engineered viral strain for GBM treatment in a murine model [118]. HSV-1, a neurotropic virus from the Herpesviridae family, is a double-stranded DNA virus. Several genetically engineered versions (e.g., HSV-1716, G207, and G47∆) of HSV-1 have been evaluated in GBM. Notably, both copies of the *RL1* gene encoding the virus protein ICP34.5 were deleted in all HSV recombinants, resulting in increased tumor selectivity [119, 120]. HSV-1716 is the first-generation HSV, and its safety has been proven in several phase I clinical trials [121–123]. The second generation of the HSV construct, G207, featuring the deletion of both copies of the *RL1* gene and an inactivating insertion of the *UL39* gene encoding infected cell protein 6 (ICP6), can generate preferential replication in dividing cells [124, 125]. The results from several clinical trials demonstrated that G207 is safe for GBM patients [126–128]. G47∆ (Teserpaturev) represents the third generation of oncolytic HSV, which is constructed on the basis of G207 with an additional deletion of the α47 gene, thus increasing viral replication and triggering antitumor immune responses via the upregulation of MHC I molecules [129]. A phase I/II trial at the University of Tokyo confirmed the safety of G47∆ in recurrent GBM patients [129, 130], followed by a phase II trial revealing its antitumor efficacy following intratumoral injections in residual or recurrent GBM [131]. As a result, G47∆ has been granted conditional time-limited approval by Japan’s Pharmaceuticals and Medical Devices Agency for brain tumor treatment. Moreover, a recent study developed a new version of G47∆ expressing IL-2 (G47Δ-mIL2), which can significantly prolong the survival of different GBM mouse models associated with increased intratumoral CD8^+^ T cells, suggesting that G47Δ-mIL2 may represent a new direction of HSV treatment for GBM patients [132], but further clinical trials are needed.

Polioviruses are positive- and single-stranded RNA viruses, and PVSRIPO is a nonpathogenic poliovirus/rhinovirus chimeric virus that targets tumor cells via the poliovirus receptor CD155, which is overexpressed in GBM tumors [133]. A phase I clinical trial (NCT01491893) demonstrated that intratumoral infusion of PVSRIPO in recurrent GBM patients was tolerated and produced increased mOS (12.5 vs. 11.3 months) and 24-month (21% vs. 14%) and 36-month (21% vs. 4%) survival rates compared with those of historical controls [134]. Currently, several clinical trials, including a phase II trial (NCT02986178) evaluating PVSRIPO as a monotherapy and phase I/II (NCT03973879) and phase II (NCT04479241) trials investigating the combination of PVSRIPO with the anti-PD-L1 antibody atezolizumab or the anti-PD-1 antibody pembrolizumab, are underway in GBM patients.

Adenoviruses are nonenveloped, double-stranded DNA viruses with icosahedral structures that have been extensively studied for cancer treatment [135]. DNX-2401 (known as tasadenoturev or Delta-24-RGD) is an engineered oncolytic adenovirus designed to selectively target and replicate in cancer cells with aberrant retinoblastoma (Rb) pathways. Specifically, DNX-2401 contains a genetic modification in the *E1A* gene, which is a 24 bp deletion responsible for Rb binding, enabling this adenovirus to replicate selectively in cancer cells with defects in the Rb pathway and decreasing virus replication in normal cells. In addition, it involves the insertion of an Arg-Gly-Asp (RGD) peptide motif, which can improve its attachment to GBM cells by binding to αV integrins. Phase I clinical trials in recurrent GBM patients demonstrated that DNX-2401 was safe and had significant antitumor effects on long-term survival in some patients [136, 137]. Furthermore, DNX-2401 in combination with TMZ was also well tolerated [138], whereas the addition of IFN-γ to DNX-2401 resulted in poor tolerability without an additional clinical benefit [139]. CRAd-S-pk7 is another glioma-tropic oncolytic adenovirus that contains the tumor-specific survivin promoter (S) and a fiber protein polylysine modification (pk7), with potential antineoplastic activity [140, 141]. Interestingly, this oncolytic adenovirus can be delivered by neural stem cells (NSCs), which can enhance the glioblastoma stem cell (GSC)-targeting ability of CRAd-S-pk7 by combining the unique tumor tropism of NSCs [142]. The safety and therapeutic potential (mOS, 18.4 months; mPFS, 9.1 months) of NSC-CRAd-S-pk7 in newly diagnosed GBM has been proven by a phase I study (NCT03072134) [143], and further clinical trials are needed to confirm its antitumor efficacy in GBM.

Moreover, adenoviruses can be modified to act as delivery vectors for exogenous genes, such as IL-12, the Fas-chimera transgene, and the HSV thymidine kinase (HSV-TK) gene. Ad-RTS-hIL-12 is an engineered adenoviral vector that delivers IL-12 controlled by the RTS promoter upon the oral administration of veledimex (VDX) [144, 145]. The tolerability and antitumor effect of Ad-RTS-hIL-12 in patients with resected recurrent GBM were evaluated in a phase I clinical trial (NCT02026271), with the results showing that peritumoral injection of Ad-RTS-hIL-12 was tolerated and resulted in an mOS of 12.7 months [146]. VB-111 is a nonreplicating adenovirus-engineered vector that can carry a Fas-chimera transgene, leading to Fas-mediated tumor apoptosis and vascular disruption [147]. In a phase I/II study (NCT01260506), combined treatment with VB-111 and bevacizumab significantly improved survival in recurrent GBM patients compared with treatment with VB-111 alone (mOS, 13.8 vs. 7.4 months; mPFS, 3.0 vs. 3.0 months) [148]. However, the subsequent phase III trial (GLOBE, NCT02511405) using combination therapy did not show a clinical benefit in recurrent GBM patients (mOS, 6.8 vs. 7.9 months; mPFS, 3.4 vs. 3.7 months) [149]. Another adenoviral vector that delivers tumoricidal gene is the HSV-TK gene, which converts prodrugs (e.g., valacyclovir, acyclovir, and ganciclovir) into toxic nucleotide analogs to kill tumor cells. The aglatimagene besadenovec (AdV-tk), a nonreplicating adenovirus expressing the HSV-TK gene, was found to be tolerated in phase I trials [150–152]. Two phase II trials demonstrated favorable PFS and OS associated with AdV-tk-based therapy in GBM patients [153, 154]. Moreover, a randomized and controlled phase III study also revealed that, compared with SOC treatment, AdV-tk gene treatment extended the survival of GBM patients (mOS, 12.9 vs. 8.6 months) [155]. However, another randomized phase III trial (ASPECT) demonstrated that sitimagene ceradenovec, another adenoviral vector used to deliver the HSV-TK gene, failed to improve survival in newly diagnosed GBM patients (mOS, 16.6 vs. 15.1 months; mPFS, 10.3 vs. 8.9 months), although it increased the time to death or reintervention [156]. Therefore, adenovirus-based delivery of HSV-TK needs to be further investigated for GBM treatment. However, a phase I study (NCT01811992) in newly diagnosed GBM patients revealed an mOS of 21.3 months when AdV-tk was combined with another adenovirus expressing FMS-like tyrosine kinase 3 ligand (Flt3L) [157].

In addition to adenovirus, the HSV-TK gene can be delivered by retroviruses. However, a phase III study revealed that retrovirus-based HSV-TK gene therapy did not generate significant clinical benefit for GBM patients compared with SOC treatment (mOS, 12.0 vs. 11.8 months; mPFS, 6.0 vs. 6.1 months) [158]. Vocimagene Amiretrorepvec (Toca 511) is another retrovirus-based treatment iteration that encodes a yeast cytosine deaminase gene and can convert the prodrug 5-fluorocytosine (5-FC) to toxic 5-fluorouracil (5-FU) [159]. Although the phase I trial resulted in encouraging efficacy data and a good safety profile [160, 161], the subsequent phase II/III trial failed to confirm the survival benefit of Toca 511/FC in GBM patients compared with SOC treatment (mOS, 11.1 vs. 12.2 months; mPFS, 6.0 vs. 6.1 months) [162]. Therefore, certain approaches (e.g., selecting the most effective virus type, optimizing the delivery method, refining genetic engineering, and combining other immunotherapy strategies) should be taken into consideration for enhancing the efficacy of virus-based therapies in GBM.

## Challenges and resistance mechanisms of immunotherapies in GBM

**Fig. 2:**
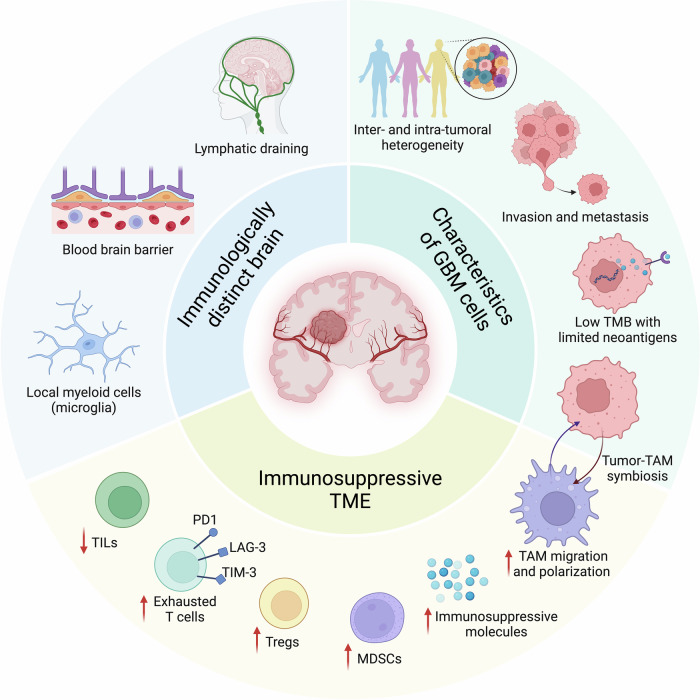
Challenges and molecular mechanisms of immunotherapy in GBM. Multiple processes restrain the response of GBM to immunotherapies, including the immunologically distinct brain (e.g., unique lympatic draining pathway, anatomical location protected by the blood‒brain barrier, and resident microglia), the immunosuppressive TME (e.g., T-cell dysfunction and exhaustion, and immunosuppressive myeloid cells, such as TAMs and MDSCs), and the characteristics of GBM cells (e.g., intertumoral and intratumoral heterogeneity, highly invasive nature, low TMB, and context-dependent GBM cells and their associated TAM biology). GBM glioblastoma, LAG-3 lymphocyte-activation gene 3, MDSCs myeloid-derived suppressor cells, PD-1 programmed cell death protein 1, TAM tumor-associated macrophage and microglia, TILs tumor-infiltrating lymphocytes, TIM-3 T-cell immunoglobulin and mucin domain 3, TMB tumor mutation burden, TME tumor microenvironment, Tregs regulatory T cells

Despite the rapid development of immunotherapy for GBM treatment, only a few strategies provide clinical benefits. Multiple processes, including the unique anatomical brain location protected by the BBB, the heterogeneity of GBM cells between and within patients, and the immunosuppressive TME, restrain immune system activity in GBM patients [163]. In this section, we summarize the challenges of immunotherapy in GBM and discuss the multiple mechanisms underlying immunotherapy resistance in GBM in detail (Fig. 2).

### “Immunologically distinct” brain

The brain has historically been considered an “immune privileged” organ, given that many studies have shown that heterotopic tissues are not rejected by animal brains, whereas the same tissues are eradicated by the host immune system when they are introduced into peripheral tissues [164, 165]. This is attributed to the lack of lymphatic drainage in the brain [166–170]. However, more studies have demonstrated that the brain engages with the immune system. Routes for antigenic egress from the brain to deep cervical lymph nodes were identified in the 1980s [167, 168, 170]. In 2015, the discovery of the presence of a dural lymphatic system within the CNS revealed a mechanism for draining CNS antigens from cerebrospinal fluid into cervical lymph nodes, facilitating immune surveillance [166, 169]. Moreover, the glial-lymphatic (glymphatic) pathway has been recently identified, providing insight into how fluids and solutes are cleared from the brain [171, 172]. Specifically, cerebrospinal fluid enters the brain along arterial perivascular spaces, engages in the interstitium through aquaporin 4 water channels, and then exits through venous perivascular spaces, draining into deep cervical and lumbar lymph nodes [172]. Therefore, the brain is an immunologically dynamic organ and should be described as “immunologically distinct” rather than “immune privileged” (Fig. 2).

The BBB, which consists of capillary endothelial cells, the basement membrane, the perivascular space, and glia limitans, is one of the inherent obstacles that impedes the entry of immune cells and immunotherapeutic drugs into the brain [173]. The BBB serves as a structural barrier that restricts the diffusion of large and hydrophilic molecules into the CNS. It also protects the brain from most blood-borne pathogens and exogenous compounds (i.e., drugs and neurotoxins) that might damage the CNS. Although the BBB is relatively impenetrable to circulating immune cells and antibodies in a quiescent state, peripheral immune cells can cross the BBB and execute vigorous inflammatory responses when danger signals are detected, providing the foundation for immunotherapy against brain tumors [174, 175]. In GBM, the BBB is partially disrupted with increased permeability due to increased angiogenesis and the release of cytokines and chemical mediators [176]. Although BBB disruption in GBM primarily occurs within the tumor core, which may enhance therapeutic delivery, the intact BBB at tumor edges can still impede the distribution of immune-related drugs [177]. To address this problem, an increasing number of studies have aimed to improve drug penetration ability by increasing drug liposolubility with liposomes or by remodeling the BBB properties via the regulation of efflux pumps and tight junctions. In addition, local administration of immunotherapeutic drugs (e.g., intrathecal drug administration, convection-enhanced delivery, and the use of implantable pharmaceutical formulations) might be another effective method for enhancing drug delivery [178]. However, randomized clinical trials are needed to evaluate the safety and effectiveness of these approaches.

Another unique immune characteristic of the brain is its resident microglia, which originate from yolk sac myeloid progenitors during development [179]. Microglia may undergo phenotypic changes in response to inflammatory stimuli [179]. Although the role of resident microglia in adaptive immunity is not fully understood, they perform a variety of critical functions, including phagocytosis, cytotoxicity, and immune regulation [179]. In GBM, resident microglia can be polarized from proinflammatory to anti-inflammatory phenotypes, which exert multiple protumorigenic activities (e.g., promoting GBM cell proliferation and invasion and stimulating angiogenesis) and inducing immunotherapy resistance. These immunosuppressive microglia inhibit effector T-cell infiltration, proliferation, and immune reactivity via distinct mechanisms, thus contributing to tumor immune evasion [180].

### Immunosuppressive tumor microenvironment

GBM is considered an immunologically ‘cold’ tumor largely due to the low number of TILs and other immune effector cell types, which are related to poor responses to immunotherapies, such as ICIs [181]. Additionally, GBM can also induce systemic immunosuppression by sequestering naïve T cells within the bone marrow, primarily through the downregulation of sphingosine-1-phosphate receptor 1, which is crucial for T-cell egress from the thymus and secondary lymphoid organs into the bloodstream [182].

Within the GBM TME, many immunosuppressive cells, such as tumor-associated macrophages and microglia (TAMs), myeloid-derived suppressor cells (MDSCs), and Tregs, together contribute to immunosuppression and GBM cell immune evasion [179, 183, 184] (Fig. 2). Among them, TAMs, which include yolk sac-derived microglia and bone marrow-derived macrophages (BMDMs), constitute the predominant cell population in the GBM TME, representing as many as 50% of the total number of cells in the entire tumor mass [179]. Recent studies demonstrated that TAMs can be educated by tumor cells, which, in turn, promote tumor progression, inhibit antitumor immunity, and induce immunotherapy resistance [34]. Tregs are a subset of T cells that contribute to tumor progression and immunotherapy resistance [185]. Specifically, cancer and microenvironmental cell-secreted IDO1, IL-10, C-C motif chemokine ligand 2 (CCL2) and transforming growth factor beta (TGF-β) promote the expansion of immunosuppressive Tregs within the GBM TME [186]. As a result, these Tregs express immune checkpoint molecules (e.g., PD-1 and CTLA-4) to suppress the effector functions of T cells [184]. MDSCs are a highly heterogeneous population of myeloid cells that contribute to tumor immunosuppression [187]. GBM cell-derived macrophage migration inhibitory factor recruits MDSCs from the bone marrow into the TME [188], and tumor-infiltrating MDSCs can suppress cytotoxic T-cell activity by expressing arginase to reduce TCR expression and induce oxidative stress to secrete reactive oxygen species [189]. Additionally, MDSCs can promote T-cell exhaustion by expressing PD-L1 [190]. Together, these findings highlight that the presence of an immunosuppressive TME represents one of the key challenges for immunotherapy in GBM.

Moreover, recent reports suggest that environmental factors, including age and sex, are associated with GBM onset, severity, and the immune response. The onset of GBM generally occurs later in life, and aging remains a major variable that can impact outcomes [191]. In relation to immunotherapies, recent work has demonstrated changes in the immune microenvironment as a result of age, leading to older individuals being more refractory to ICIs [192]. In addition to age, sex is a major biological variable that impacts GBM growth and therapeutic resistance. From an epidemiological standpoint, males develop GBM more frequently [193] and have poorer outcomes [194]. A recent clinical report also revealed sex differences in MGMT methylation, where a survival advantage was observed only in females [195]. While these differences can be attributed to different signaling networks [196], recent work in preclinical models and human samples has shown that male T cells are more prone to exhaustion and more responsive to ICIs [197], whereas MDSC subset localization is sex biased, with males having an enrichment of monocytic MDSCs in the TME, whereas females having increased granulocytic MDSCs in their circulation [198]. Moreover, these differences were also leveraged for preclinical validation of sex-specific immunotherapies [198]. These observations further support earlier work in which sex differences in microglia also impacted GBM growth [199, 200]. Taken together, age and sex are emerging as key biological variables in GBM and have implications for the GBM immune response and immunotherapy strategies.

### Unique GBM cell characteristics

In addition to the immunosuppressive TME, the biological activities of cancer cells also contribute to immunosuppression and immunotherapy resistance in GBM (Fig. 2). First, the complexity and heterogeneity of GBM cells pose significant challenges for immunotherapies. GBM tumors exhibit both intertumoral and intratumoral heterogeneity, impacting their different responses to immunotherapies on the basis of molecular profiles, such as dysregulated p53, Rb, and phosphoinositide 3-kinase pathways [201]. In addition to interpatient heterogeneity, intratumoral variability further increases the difficulty of immunotherapy in GBM. For example, the individual tumor mass harbors a complex and dynamic architecture of tumor cells, exhibiting variability at the epigenetic, transcriptomic, protein, and metabolic levels [202, 203]. Moreover, treatment may induce GBM tumor phenotype switching, and relapsed GBM tumors might have different tumor subtypes and accessible targets than newly diagnosed tumors do [204]. Therefore, developing immunotherapy approaches that specifically target tumor cell subpopulations might be a new direction for GBM treatment.

Second, the highly invasive capacity of GBM cells might contribute to tumor recurrence and treatment resistance [205]. The different invasive potentials of GBM cells increase their intratumoral heterogeneity. Specifically, cells in the tumor core exhibit heightened proliferative capacity, whereas those at the tumor periphery are more prone to infiltration, enabling them to penetrate surrounding normal brain tissues [205]. After infiltrating brain tissues, GBM cells remodel the extracellular matrix, cytoskeleton, and metabolism [206]. Although GBM cells rarely metastasize to distant organs [207], this invasion and infiltration decrease the possibility of complete surgical resection and lead to a high chance of tumor recurrence even after multiple postoperative adjuvant therapies, including immunotherapy.

Third, GBM cells exhibit a low tumor mutation burden (TMB) and consequently have limited neoantigen presentation for effective T-cell recognition [208, 209]. This is primarily because the presentation of neoantigens depends on the abundance of mutations capable of generating neoepitopes [210]. Although some mutations can be immunogenic and presented by APCs, the majority of mutations do not become MHC-presented neoepitopes that can be recognized and targeted by T cells [211]. Moreover, tumor subclones without highly antigenic peptides might evade immune surveillance [211]. The low TMB-induced smaller antigen pool allows fewer immunogenic neoantigens to be exposed following immunotherapy, which increases the challenges of immunotherapy in GBM.

**Fig. 3:**
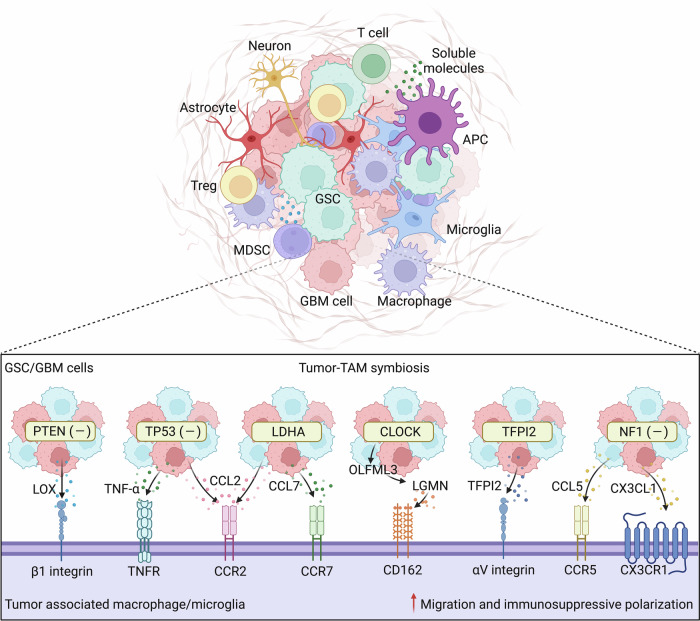
The immunosuppressive TME and tumor-TAM crosstalk in GBM. The immunosuppressive TME of GBM is a highly heterogeneous dynamic system that includes tumor cells (GBM cells and GSCs), low numbers of TILs, high infiltration of immunosuppressive cells (e.g., TAMs, MDSCs, and Tregs), normal brain cells (e.g., astrocytes and neurons), and soluble molecules. Crosstalk between tumor cells and TAMs is an important mechanism within the GBM TME that promotes tumor growth and induces immunosuppression. Under different conditions (e.g., PTEN, P53 and NF1 deletion/mutation, CLOCK and TFPI2 overexpression/amplification, and metabolic changes), tumor cells can secrete various cytokines and chemokines, such as LOX, TNF-α, OLFML3, LGMN, TFPI2, CCL2, CCL5, CCL7, and CX3CL1, to promote the migration and immunosuppressive polarization of TAMs, which in turn promotes tumor progression and immunosuppression. APCs antigen-presenting cells, CCL2 C-C motif chemokine ligand 2, CD162 cluster of differentiation 162, CLOCK circadian locomotor output cycles kaput, CX3CL1 C-X3-C motif ligand 1, CX3CR1 C-X3-C motif chemokine receptor 1, GBM glioblastoma, GSCs glioblastoma stem cells, LDHA lactate dehydrogenase A, LOX lysyl oxidase, LGMN legumain, MDSCs myeloid-derived suppressor cells, NF1 neurofibromin 1, OLFML3 olfactomedin like 3, TAMs tumor-associated macrophages and microglia, TFPI2 tissue factor pathway inhibitor 2, TILs tumor infiltrating lymphocytes, TME tumor microenvironment, TNF-α tumor necrosis factor α, TNFR, CCR2 C-C motif chemokine receptor 2, TNFR TNF-α receptor, Tregs regulatory T cells

Fourth, GBM cells can express immunosuppressive molecules, such as PD-L1, IDO1, IL-10 and TGF-β, to cause the dysfunction and exhaustion of TILs [212]. For example, TGF-β secreted by GBM cells decreases the expression of the activated receptor natural killer group 2D on CD8^+^ T cells and NK cells, thereby reducing their cytotoxic effects on GBM cells [213]. Moreover, tumor cells also decrease their MHC expression levels to reduce the likelihood of tumor antigen presentation [214]. On the other hand, context-dependent signaling from GBM cells can affect myeloid cell biology, such as TAM migration, polarization, and activation (Fig. 3), generating context-dependent tumor-TAM symbiotic interactions to promote GBM progression and immunosuppression [212, 215]. For example, *PTEN* deletion/mutation in GBM cells increases the expression and secretion of lysyl oxidase to promote macrophage infiltration via activation of the β1 integrin-protein-tyrosine kinase 2 signaling axis in macrophages [216]. *TP53* gain-of-function mutation leads to the upregulation of CCL2 and tumor necrosis factor α (TNF-α) via nuclear factor-kappa B (NF-κB) signaling, which, in turn, increases the infiltration of microglia and monocyte-derived immune cells [217]. In addition to genetic alterations, metabolic changes resulting from lactate dehydrogenase A (LDHA) overexpression in GBM cells increase CCL2 and CCL7 expression to attract macrophages into the TME [218]. Eventually, these GBM-associated TAMs are polarized toward an immunosuppressive phenotype, thus inhibiting T-cell-mediated antitumor immunity and inducing immunotherapy resistance [216–218].

Finally, GSCs constitute a subpopulation of GBM cells that are characterized by stem cell-like capabilities, which drive tumor self-renewal and clonal tumor initiation, eventually promoting intratumoral heterogeneity and therapeutic resistance [219]. While GSCs account for only a small fraction (~10%) of the total GBM cells within tumor tissues, a large population of proliferative cancer cells are composed of GSCs [219]. GSCs can escape immune surveillance by recruiting immunosuppressive PD-L1^+^ macrophages [220]. Crosstalk between GSCs and TAMs is an important mechanism within the GBM TME that promotes the immune escape of GBM tumors [212, 215]. For example, a gain-of-function screen of epigenetic regulators identified circadian locomotor output cycles kaput (CLOCK, a gene amplified in approximately 5% of GBM cases) as a key hit in GSCs that not only promotes stemness but also triggers microglial infiltration and immunosuppressive polarization by transcriptionally upregulating olfactomedin-like 3 and legumain (LGMN). As a result, these microglia promote tumor growth and suppress antitumor immunity [221, 222]. The Kunitz-type protease inhibitor TFPI2 is another example that is amplified in approximately 4% of GBM cases and can regulate GSC–microglia interactions. Specifically, TFPI2 is highly expressed in GSCs, where it not only promotes GSC self-renewal and tumor growth but also triggers microglial infiltration and immunosuppressive polarization to induce immunosuppression [223]. Moreover, neurofibromin 1 (Nf1)-deficient GSCs isolated from tumors of the *Nf1* genetically engineered mouse model can produce the chemokines C-X3-C motif ligand 1 and CCL5 to recruit microglia into the TME [224], which ultimately exhibit an immunosuppressive function.

## Combinational strategies to overcome immunotherapy resistance

**Fig. 4:**
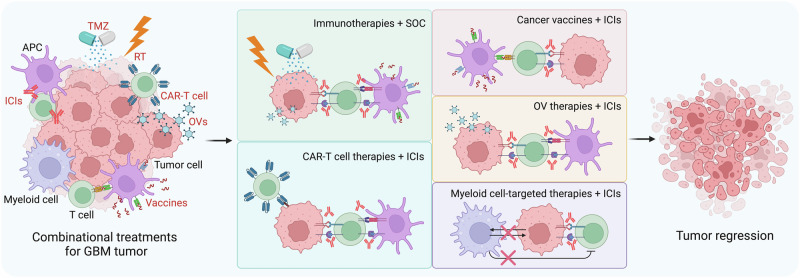
Strategies for overcoming immunotherapy resistance in GBM. Multiple combination strategies, including SOC combined with immunotherapies, ICIs combined with other types of immunotherapies (e.g., CAR-T-cell therapies, cancer vaccines, and OV therapies), and myeloid cell-targeted therapies combined with ICIs, have been developed to overcome immunotherapy resistance in GBM. APC antigen-presenting cell, CAR chimeric antigen receptor, GBM glioblastoma, ICIs immune checkpoint inhibitors, OV oncolytic virus, RT radiotherapy, SOC standard of care, TMZ temozolomide

Current observations suggest that a single immunotherapeutic approach is ineffective for GBM, which is highly complex and heterogeneous, as described above. GBM may develop various resistance mechanisms for monoimmunotherapy. For example, tumor vaccines and CAR-T cells can lead to the continuous loss of tumor antigens and increased numbers of inhibitory cells and factors in the TME [66, 77]. The efficacy of ICIs is also limited by the immunosuppressive TME and low immunogenicity of GBM cells [28]. Given the multiple mechanisms that mediate monoimmunotherapy resistance, combining immune-based approaches with SOC or other immune-remodeling strategies represents a new direction for GBM treatment and has the potential to overcome immunotherapy resistance. In this section, we discuss distinct combinational strategies that have the potential to overcome immunotherapy resistance in GBM (Fig. 4).

### Standard of care in combination with immunotherapies

The combination of immunotherapies with chemotherapy, particularly TMZ, is one of the most investigated strategies for GBM treatment. A phase II study in newly diagnosed GBM patients demonstrated that combining the anti-PD-1 antibody pembrolizumab with TMZ and tumor treatment resulted in an mPFS of 12.0 months compared with 5.8 months in case-matched controls [225]. However, the adjuvant anti-CTLA4 antibody ipilimumab combined with TMZ after surgery and chemoradiotherapy did not result in a survival benefit for newly diagnosed GBM patients (mOS, 22.7 vs. 26.4 months; mPFS, 10.9 vs. 12.5 months) [42]. IDO1-targeted ICI therapy is being investigated in combination with TMZ and RT in newly diagnosed GBM patients (NCT04047706 and NCT02052648). Moreover, other immunotherapeutic strategies, including the peptide vaccine SurVaxM [83, 84], the DC vaccine DCVax-L [100], and the oncolytic adenovirus DNX-2401 [138], in combination with TMZ are being evaluated in clinical trials, and some of them have already shown encouraging results in GBM patients.

RT can cause molecular damage (e.g., DNA breaks and base modifications) and trigger immunogenic cell death in GBM cells [226]. With respect to the immune response, RT can promote the release of tumor neoantigens and inflammatory cytokines, recruit effector T cells to the tumor site, and activate the cyclic GMP-AMP synthase (cGAS)-stimulator of interferon genes (STING) pathway, laying the foundation for RT in combination with immunotherapies [226]. Recently, several clinical trials have been designed to explore the safety and efficacy of such combination therapy. For example, a phase I study demonstrated that combination treatment with hypofractionated RT (5 × 6 Gy), the anti-PD-1 antibody pembrolizumab, and the anti-VEGF antibody bevacizumab was well tolerated in GBM patients and induced promising survival results (mOS, 13.4 months; mPFS, 7.9 months) [227]. Further clinical studies are needed to explore the optimal dose and fractionation scheme of this combination strategy for GBM patients.

Although the development of a therapeutic approach that combines immunotherapy with SOC is promising, some concerns and associated solutions should be taken into consideration. First, systemic TMZ chemotherapy could cause a reduction in lymphocytes, and for this reason, local TMZ treatment might be a better administration approach when combined with ICIs [228]. Second, patients whose MGMT promoter is not methylated are resistant to TMZ treatment [229], and MGMT promoter methylation may be considered a biomarker for patient selection. Third, immunotherapy efficiency is affected by the dose and treatment timing, which should be determined before clinical trials. Together, the combination of immunotherapy with SOC shows great therapeutic potential, and further clinical trials are needed to validate the efficiency of such a combination treatment strategy in GBM patients.

### Immune checkpoint inhibitors in combination with other immunotherapies

Although ICIs are widely used to treat a variety of cancers, such as melanoma and non-small cell lung cancer, the efficacy of ICIs in GBM patients is still disappointing [163, 230]. One of the key reasons for their ineffectiveness is the low immunogenicity of GBM with a relatively low number of TILs. Indeed, the efficacy of ICIs in GBM is highly dependent on the abundance and activation status of TILs, especially T cells [28]. Therefore, other immunotherapeutic approaches aimed at increasing T-cell infiltration and activation, such as CAR-T-cell therapy, cancer vaccines, and OV therapy, might be potential candidates for overcoming ICI resistance in GBM. Here, we discuss the therapeutic potential of combining ICIs with other types of immunotherapies for GBM treatment (Fig. 4).

#### Combination of CAR-T-cell therapies with immune checkpoint inhibitors

CAR-T-cell therapy, a breakthrough in cancer treatment, has broadened the immunotherapy landscape [55]. However, antigen loss, immunoediting, and adaptive resistance are common phenomena that typically occur after CAR-T-cell therapy [66, 231]. In addition, increased immunosuppression (e.g., upregulation of PD-1 expression) is always accompanied by CAR-T-cell therapy [232, 233]. Therefore, combining CAR-T-cell therapy with other treatment modalities (e.g., ICIs) might be an efficient way to overcome the immunoresistance associated with a single treatment [234]. The clinical exploration of combining CAR-T-cell therapy with ICIs for GBM treatment is still in the early stage. A recent phase I clinical trial (NCT03726515) revealed that the combination of EGFRvIII-CAR-T-cell therapy with anti-PD-1 therapy, pembrolizumab, did not provide clinical benefit for newly diagnosed and EGFRvIII^+^ GBM patients (mOS, 11.8 months; mPFS, 5.2 months), although the combination strategy was well tolerated in patients [235]. Another phase I clinical trial (NCT04003649) combining IL-13Rα2 CAR-T-cell therapy with or without anti-PD-1 therapy, nivolumab, and anti-CTLA-4 therapy, ipilimumab, is underway, highlighting the potential for GBM treatment via this new combination treatment strategy. Furthermore, engineered CAR-T cells expressing immune checkpoint antibodies (e.g., CAR-T cells expressing anti-PD-1 and anti-CTLA-4 mAbs) may represent a new direction for GBM treatment, and several clinical trials (e.g., NCT02873390, NCT02937844, and NCT03182816) are ongoing.

#### Combination of cancer vaccines with immune checkpoint inhibitors

Cancer vaccines can increase antitumor immunity by increasing immunogenicity and activating peripheral T cells, which is important for increasing the antitumor efficacy of ICIs [236]. Moreover, the increased expression of immune checkpoints (e.g., PD-L1) after cancer vaccine therapy provides a further theoretical basis for the combination of cancer vaccines with ICIs [77], which has been found to benefit GBM patients [237]. DC vaccines have shown promising results when combined with ICIs in GBM patients. A two-arm randomized trial in recurrent GBM patients (NCT02529072) demonstrated that treatment with a DC vaccine in combination with the anti-PD-1 agent nivolumab resulted in prolonged survival compared with single nivolumab treatment (mOS, 15.3 vs. 8.0 months; mPFS, 6.3 vs. 4.3 months). Moreover, a recent case report revealed that cotreatment with DC vaccines, anti-PD-1, and poly I:C after SOC exhibited profound antitumor efficacy, and the patient remained disease free for 69 months [238]. Another clinical trial combining the DC vaccine autologous tumor lysate-pulsed DC with the anti-PD-1 agent pembrolizumab in progressive and recurrent GBM patients is ongoing (NCT04201873). In addition to DC vaccines, other cancer vaccines have also been used in combination with ICIs. A recent phase II clinical trial (NCT03018288) evaluated the safety and antitumor efficacy of combining the peptide vaccine HSPPC-96 with anti-PD-1, pembrolizumab, and chemoradiotherapy in newly diagnosed GBM patients, but no clinical benefits were observed. The other exciting progress in the field includes current efforts aimed at testing the clinical benefits of a treatment strategy that combines novel tumor vaccines (e.g., personalized vaccines and DNA/RNA vaccines) with ICIs in GBM patients (NCT02287428, NCT05743595, NCT03665545 and NCT03491683).

#### Combination of oncolytic viral therapies with immune checkpoint inhibitors

OVs are designed to induce immunogenic cell death through direct virus-mediated cytotoxicity in cancer cells, thus releasing tumor antigens and proinflammatory cytokines [117]. OV therapies can not only “heat up” the GBM TME by recruiting and activating TILs but also increase PD-L1 expression in tumors to sensitize them to ICIs [239]. Therefore, combining OV therapies with ICIs is a promising strategy for GBM treatment. Among OVs, adenoviruses have been mostly used in combination with ICIs. A phase I/II clinical trial (NCT02798406) evaluated the antitumor efficiency of the oncolytic adenovirus DNX-2401 combined with the anti-PD-1 antibody pembrolizumab in recurrent GBM patients [240]. Compared with the prespecified control, the combination treatment regimen led to a significantly greater 12-month OS rate (52.7% vs. 20%). Ad-RTS-hIL-12 is an adenovirus engineered with an IL-12 transgene controlled by the RTS promoter, and the results from a phase I trial (NCT03636477) revealed that the combination of Ad-RTS-hIL-12 and the anti-PD-1 agent nivolumab was well tolerated in recurrent GBM patients and resulted in an mOS of 16.9 months [241]. These encouraging results lead to an ongoing phase II clinical trial (NCT04006119) evaluating the efficacy of this combination strategy in recurrent or progressive GBM patients. AdV-tk is another excellent example of an adenovirus suitable for combination with ICIs to treat GBM. A preclinical study demonstrated that combination treatment with AdV-tk and an anti-PD-1 antibody increased intratumoral T-cell infiltration and prolonged the survival of GBM-bearing mice [242]. Encouraged by these preclinical findings, a phase I clinical trial (NCT03576612) evaluating the combination strategy (AdV-tk combined with nivolumab) in GBM patients is underway. In addition to adenoviruses, the antitumor effects of the poliovirus PVSRIPO combined with or without the anti-PD-1 agent pembrolizumab (NCT04479241) or the anti-PD-L1 agent atezolizumab (NCT03973879) in GBM patients are currently being evaluated in two ongoing phase I/II trials.

### Combinations of myeloid cell-targeted therapies with immune checkpoint inhibitors

Myeloid cells play crucial roles in regulating GBM progression and immunosuppression and inducing immunotherapy resistance [179, 243]. GBM is considered a “cold” tumor infiltrating high levels of immunosuppressive myeloid cells [212]. TAMs constitute the largest population of myeloid cells in the GBM TME, where their infiltration and immunosuppressive polarization are usually triggered by factors secreted by GBM cells [179]. As a result, these polarized TAMs contribute to several GBM tumor hallmarks, including immunosuppression [215]. Blockade of TAM infiltration and immunosuppression markedly suppresses tumor progression and activates antitumor immunity in GBM mouse models [218, 221–223]. These findings suggest that targeting TAMs is a promising antitumor strategy that has the potential to affect ICI therapy efficacy in GBM. Here, we discuss advances in therapeutic strategies that combine myeloid cell-targeted therapies with ICIs in GBM.

The cGAS-STING pathway serves as a sensor of cellular stress that can activate innate immunity and antigen presentation by myeloid cells and is recognized as the most promising therapeutic target for GBM treatment [244–246]. Indeed, STING agonists function as potential therapeutic drugs to influence the TME, including the infiltration of inflammatory macrophages and neutrophils and the upregulation of PD-L1 [247]. As a result, the combination of the clinical-grade STING agonist 8803 with an anti-PD-1 antibody markedly enhances the survival of GBM tumor-bearing mice [248]. Histone 3 lysine 27 demethylase (KDM6B) is an epigenetic enzyme that is highly expressed in immunosuppressive myeloid cells (e.g., immunosuppressive macrophages). Inhibition of myeloid cell KDM6B genetically (using myeloid cell *Kdm6b*-specific knockout mice) or pharmacologically (using the KDM6B inhibitor GSK-J4) sensitizes GBM-bearing mice to anti-PD-1 therapy [249]. LGMN is a protease that is highly enriched in TAMs. Inhibition of LGMN genetically and pharmacologically enhances T-cell-mediated antitumor immunity and synergizes with anti-PD-1 therapy in GBM tumor-bearing mice [250]. In addition to LGMN, sialic acid binding Ig like lectin 9 (SIGLEC9), which is expressed in a unique immunosuppressive macrophage subpopulation from GBM patients who do not respond to anti-PD-1 treatment, has been identified. Deletion of SIGLEC9 in GBM mouse models synergizes with anti-PD-1 or anti-PD-L1 therapy [251]. Similarly, MDSC-targeted therapies have the potential to improve the antitumor efficacy of ICIs. For example, C-X-C motif chemokine receptor 4 (CXCR4) is commonly overexpressed in tumor-associated MDSCs, and anti-CXCR4 therapy can synergize with anti-PD-1 therapy to increase the survival of GBM tumor-bearing mice [252]. Together, these findings suggest that strategies that target myeloid cells can increase the antitumor efficacy of ICIs in GBM mouse models.

Emerging evidence indicates that the symbiotic interaction between GBM cells and myeloid cells is critical for immunotherapy resistance [212, 253]. On the basis of the understanding and progress in the field, here, we summarize recent findings highlighting the therapeutic potential of targeting tumor–TAM symbiosis to increase the effectiveness of ICIs in GBM. For example, the inhibition of GSC–microglia symbiosis via the targeting of CLOCK and its downstream CD162 can activate antitumor immunity and synergize with anti-PD-1 therapy in GBM mouse models [221]. TFPI2 is another key mediator that can modulate GSC–microglia interactions. Blockade of this symbiosis by genetic depletion of TFPI2 in GSCs or pharmacologic inhibition of the TFPI2 receptor CD51 and its downstream STAT6 in microglia activates antitumor immunity and enhances the therapeutic efficiency of anti-PD-1 therapy in animal models of GBM tumors [223]. In addition to these newly identified strategies, we recently reviewed the accumulating approaches that can target tumor-TAM symbiosis to improve the effectiveness of ICIs in GBM [212, 253]. Although these findings highlight the importance and therapeutic potential of targeting myeloid cells and/or tumor-myeloid cell symbiosis to improve the effectiveness of ICIs in GBM, the current knowledge in this field is still at an early stage, and clinical trials are needed to validate this combination therapeutic strategy in GBM patients.

## Conclusions and perspectives

GBM is the most aggressive and malignant form of glioma, with an mOS of only 12–18 months [3]. With the revolutionary success of immunotherapy in multiple cancer types, such as FDA-approved pembrolizumab and nivolumab for treating non-small cell lung cancer and melanoma, an increasing number of immunotherapies, including ICIs, adoptive T-cell therapies, tumor vaccines, and OV therapies, have been tested in preclinical and clinical studies for GBM [254]. However, the area of immunotherapy in GBM is facing enormous challenges, and the majority of phase III clinical trials have failed to yield clinically beneficial outcomes. The high failure rate of clinical trials highlights the urgent need to better understand GBM biology, including the unique features of the brain, GBM cell biology, the immunosuppressive TME, and tumor-TME symbiosis [181, 255].

The unique immune features of the brain, including the immunologically quiescent state, the function of the BBB, and the population of resident myeloid cells, contribute to the distinct immunological nature of GBM tumors, raising challenges for immunotherapies [4]. Tumor heterogeneity, including both intertumoral and intratumoral heterogeneity, is another important factor in the failure of immunotherapies. Novel approaches, such as single-cell RNA sequencing and spatial transcriptomics, may help to identify potential powerful immunotherapeutic targets for GBM [256, 257]. Furthermore, the highly immunosuppressive TME accounts for the ineffectiveness of current immunotherapies for GBM patients. Together, the low numbers of TILs and high infiltration of immunosuppressive cells (e.g., TAMs, MDSCs and Tregs) contribute to the immune evasion of GBM cells. Importantly, the interactions between tumor cells and myeloid cells (e.g., tumor-TAM symbiosis) further promote immune escape and immunotherapy resistance in GBM. Many preclinical studies have demonstrated that inhibition of tumor-TAM symbiosis can significantly prolong the survival of GBM tumor-bearing mice and synergize with ICIs, especially anti-PD-1 antibodies [212, 221, 223, 253, 258]. Finally, recent findings of the impact of age and sex may also influence the immune response and outcome and should be integrated into both preclinical studies and powered appropriately in clinical trials. Taken together, additional studies are needed to explore immunotherapy resistance mechanisms in depth and translate these preclinical findings into clinical settings.

Current immunotherapies focus mainly on the effector arm of the immune system, such as reinvigorating the T-cell response by blocking immune checkpoints, using tumor vaccines to activate adaptative immune responses, or directly transferring engineered T cells into the tumor. However, such treatment may not provide optimal results, as certain tumors, including GBM, have a relatively low number of TILs in the TME. Moreover, these lymphocytes can develop into an exhausted state after therapy [259]. Therefore, more attention should be given to enhancing the infiltration of TILs and blocking the function of immunosuppressive factors in the TME. Furthermore, given the inefficiency of a single immunotherapeutic agent, combination treatment, such as combination with SOC or other different immune-based therapies, might be a potential strategy to overcome the immunotherapy resistance observed in GBM patients. Efforts focused on developing combinational therapies and other novel strategies to turn the ‘cold’ GBM TME into a ‘hot’ TME have increasingly been recognized in both preclinical and clinical studies. Novel strategies that simultaneously target innate and adaptive immunity might be an efficient way to achieve better tumor clearance. In addition, identifying novel biomarkers that can predict which population of GBM patients can respond better to specific types of immunotherapies is critical. Given tumor heterogeneity, context-dependent biomarkers and treatment strategies might lead to maximum clinical benefit for GBM patients. Overall, despite many obstacles, immunotherapy is still one of the most promising therapeutic approaches for GBM, and developing novel combinational therapeutic strategies aimed at “heating up” GBM tumors might overcome immunotherapy resistance.
